# Characteristics of Anemia in Children Aged 6 Months to 5 Years Attending External Consultations at a Pediatric Hospital in Lisbon, Portugal

**DOI:** 10.3390/children12070832

**Published:** 2025-06-24

**Authors:** Réka Maulide Cane, Sérgio Chicumbe, Youssouf Keita, Anaxore Casimiro, Bárbara Martins Saraiva, Joana Vasconcelos, Beatriz Luzio Vaz, Afonso Sousa, Mafalda Cabral, Francisco Branco Caetano, Luís Varandas, Isabel Craveiro

**Affiliations:** 1 Instituto Nacional de Saúde, INS, Ministério da Saúde, MISAU, Estrada Nacional EN1, Bairro da Vila—Parcela no 3943, Distrito de Marracuene, Marracuene 264, Província de Maputo, Mozambique; sergio.chicumbe@ins.gov.mz; 2 Global Health and Tropical Medicine, GHTM, Associate Laboratory in Translation and Innovation Towards Global Health, LA-REAL, Instituto de Higiene e Medicina Tropical, IHMT, Universidade NOVA de Lisboa, UNL, Rua da Junqueira 100, 1349-008 Lisbon, Portugal; anaxore.casimiro@ulssjose.min-saude.pt (A.C.); varandas@ihmt.unl.pt (L.V.); isabelc@ihmt.unl.pt (I.C.); 3 World Bank, Global Financings Facility, Bamako, Mali; ykeita@worldbank.org; 4 Hospital Dona Estefânia, Unidade Local de Saúde São José, 1169-045 Lisbon, Portugal; barbara.saraiva@ulssjose.min-saude.pt (B.M.S.); joanavasconcelos@ulssjose.min-saude.pt (J.V.); ana.vaz3@ulssjose.min-saude.pt (B.L.V.); afonso.sousa@ulssjose.min-saude.pt (A.S.); mafalda.cabral@ulssjose.min-saude.pt (M.C.); francisco.caetano@ulssjose.min-saude.pt (F.B.C.); 5 NOVA Medical School, Faculdade de Ciências Médicas, Universidade Nova de Lisboa, Campo Mártires da Pátria 130, 1169-056 Lisbon, Portugal

**Keywords:** childhood, anemia, characteristics, Portugal, Southern Europe, Europe

## Abstract

**Background/Objectives:** Childhood anemia remains a serious public health issue, negatively affecting cognitive and psychomotor development, with repercussions on school performance and adult productivity. This study aimed to characterize the profile of children aged 6 months to 5 years diagnosed with or at risk of anemia who attended a pediatric hospital in Lisbon, Portugal. **Methods:** A hospital-based, cross-sectional descriptive study was conducted from September 2023 to September 2024. Descriptive statistics, including frequency distributions and cross-tabulations, summarized participant characteristics and key variables. **Results:** We observed that 33.3% (74/222) of the children were either anemic or at risk of anemia. Among these, 93.2% (69/74) were confirmed anemic or at risk based on hemoglobin levels. Five children (6.8%) had normal hemoglobin but abnormal red-cell indices, with microcytic (60.0%; 3/5) or normocytic (40.0%; 2/5) patterns. Anemia rates were higher in males (55.1%), children aged 24–59 months, those residing in the Metropolitan Lisbon Area (82.6%), children whose caregivers had only basic or secondary education (58.0%), and those whose mothers were born in foreign countries (48.4%). Microcytic red-cell indices were observed in 63.1% of cases. Serum iron results indicated that 32.0% were pre-anemic and 40.0% anemic. Ferritin levels showed iron-deficiency anemia in 22.2% of tested cases. In addition, 33.3% carried the sickle cell trait, and 35.0% had elevated C-reactive protein, suggesting anemia of inflammation. **Conclusions:** Anemia is a moderate public health issue, mainly affecting children with less-educated caregivers and migrant mothers. Targeted public health actions, including systematic screening, caregiver education, and multiculturally sensitive interventions, are crucial to address anemia.

## 1. Introduction

Anemia is a condition where the number of erythrocytes or red blood cells (RBCs) is insufficient to meet metabolic demands [1]. It is a serious public health problem that affects both high- and low-income countries and is associated with adverse health outcomes and reduced quality of life [1,2,3,4,5,6,7]. Globally, anemia is one of the most widespread nutritional disorders, affecting nearly 40.0% of children aged 6 months to 5 years [3,7,8,9].

The causes of childhood anemia are multifactorial. For example, lack of iron, folate, vitamin B12, and vitamin A can cause nutritional anemia [6,7,9]. Iron deficiency is the most common nutritional deficiency. It often occurs when dietary intake is insufficient to meet physiological demands, particularly during life stages when requirements are elevated, such as pregnancy, infancy, and periods of rapid growth and development [10,11].

Deficiencies in several other micronutrients (e.g., vitamin A, B12, folic acid, and riboflavin), which are essential for the normal production of red blood cells, likely contribute to the development of anemia [11]. Nutritional and iron deficiency anemia in children impairs immune function, delays mental, physical, and socioemotional development, and increases the risk of death among infants and young children [7,9]. Non-nutritional causes of anemia include parasitic infections (e.g., malaria, hookworm, schistosomiasis), as well as blood loss, blood-inherited diseases (e.g., thalassemia and sickle-cell), and infectious diseases (e.g., HIV/AIDS) [7,9]. The interaction between anemia and infections is complex: anemia may increase susceptibility to infections, while infections can contribute to anemia through mechanisms such as inflammation, hemolysis, and nutrient malabsorption [12,13]. Parasitic infections impair the intestinal absorption of nutrients, often through the consumption of contaminated water or food, leading to anemia [14]. In addition, sociodemographic and economic factors, child feeding practices, access to health services, and maternal anemia also play a role in the development of childhood anemia [15,16]. Anemia represents a key indicator of poor health and nutrition, acting as a marker of socioeconomic disparities, with children from lower-income households at higher risk [17]. Poverty, limited access to healthcare services, and low caregiver education levels restrict access to nutritious foods and timely medical interventions, thereby increasing the risk of childhood anemia [18]. Few studies [19,20,21] have also shown an association between maternal and fetal anemia.

Anemia is defined quantitatively by hemoglobin (Hb), hematocrit, or red blood cell count levels that fall below the normal age- and sex-specific ranges [1]. Anemia in children aged 6 months to 5 years is characterized by low blood hemoglobin and can be classified into mild, moderate, and severe categories [6,11,22,23]. Anemia symptoms vary according to severity, with mild cases often being asymptomatic and severe cases associated with developmental impairments and increased childhood mortality [16,24,25,26,27]. Anemia is also classified by the mean corpuscular volume (MCV) into microcytic, normocytic, and macrocytic types [1,28,29]. Microcytic anemia is often caused by iron deficiency or thalassemia [30,31,32]. Normocytic anemia may result from chronic infections, systemic diseases, and acquired disorders (e.g., autoimmune hemolytic anemia and microangiopathic hemolysis) [1,31,33]. The premature breakdown of red blood cells that occurs in hemolytic anemia may also increase bilirubin levels [1]. Macrocytic anemia is usually caused by deficiencies in vitamin B12 or folic acid, but it may also result from chronic liver disease, hypothyroidism, myelodysplastic disorders, and bone marrow disorders such as leukemia [1,30,31,34,35].

In 2021, the global anemia prevalence was 24.3%, affecting 1.92 billion people and accounting for 52.0 million years of healthy life lost due to disability (YLDs) [36]. In children aged 6–59 months, the global prevalence was 40.0% in 2019, a modest decline from 48.0% in 2000 [37]. The highest burden remains in Africa, where 60.2% of children under five are affected, compared to 23.0% in Europe [36,37,38,39]. In Portugal, childhood anemia data are scarce. National estimates from 2019 suggest a prevalence of 4.3% in children under five [40], though other studies report that anemia is highly prevalent and largely undiagnosed [41,42]. While studies on childhood anemia in Portugal have been conducted, few were inherently review articles [43,44]. A 2005 study in Braga reported a 19.7% prevalence of iron deficiency anemia in infants [45], but more recent research is limited [46]. Given the implications of anemia on development and productivity, there is an urgent need for updated research. This study aims to characterize the profile of children aged 6 months to 5 years diagnosed with or at risk of anemia attending external pediatric consultations at Dona Estefânia Hospital (HDE) in Lisbon, Portugal. It seeks to provide updated epidemiological evidence to inform public health strategies and improve the management of childhood anemia.

## 2. Materials and Methods

### 2.1. Study Setting

Portugal, officially the Portuguese Republic, is the westernmost country in mainland Europe [47,48,49]. This study was conducted at the External Consultations of the Pediatrics Medical Service of Dona Estefânia Hospital (HDE)—Unidade Local de Saúde de São José (ULS São José) in Lisbon. Dona Estefânia Hospital is a reference pediatric center for Southern Portugal and the archipelagos of the Azores and Madeira. It specializes in maternal and child health care [50,51]. In 2024, Dona Estefânia Hospital recorded a total of 59,870 pediatric consultations across all medical specialties. Of these, 4169 were in general pediatrics, with 1046 corresponding to first consultations and 2420 to follow-up consultations, covering a total of 2301 patients [51].

### 2.2. Study Design, Population, and Sample Universe

This hospital-based, cross-sectional descriptive study was conducted from September 2023 to September 2024. The sample universe was the External General Pediatrics Consultation section (area) of the Dona Estefânia Hospital. During the study period, all children aged 6 months to 5 years who attended the external general pediatric consultations were recorded (census-based approach). From this population, only those meeting the inclusion criteria—children diagnosed with or at risk of anemia—were included in the final study sample.

### 2.3. Inclusion and Exclusion Criteria

The inclusion criteria were a subset of children aged between 6 months and 5 years, specifically those diagnosed with or at risk of anemia, who attended the External General Pediatrics Consultation section (area) at Dona Estefânia Hospital. This included children referred from the emergency department for evaluation or follow-up by a pediatrician; children referred by family doctors from Health Centers; and children evacuated from Portuguese-speaking African countries due to various pathologies not treatable in their countries of origin. Children with normal hemoglobin levels but altered red-cell indices, including microcytic and normocytic patterns, were also included to capture potential subclinical anemia cases.

The exclusion criteria were children younger than 6 months, children aged 6 years or older, and children without available hemoglobin or hematocrit measurements. Children being followed in hematology or other specialized consultations, as well as those with pre-existing hematologic or cardiovascular diseases, were excluded from this study.

### 2.4. Data Collection and Quality Control

The total number of children aged 6 months to 5 years, both with and without anemia, who attended external pediatric consultations during the study period was obtained from daily consultation records. Children diagnosed with anemia or those identified as at risk were selected during each consultation. The study was then explained to the children’s caregivers, and their written informed consent was obtained. A pretested questionnaire, consisting of three sections on sociodemographic characteristics, feeding habits, and health status, was used to collect information. Clinical data, including laboratory test results, were obtained from electronic medical records and children’s health cards. Typically, all children had a complete blood count (CBC) performed. Additional tests—such as serum iron, ferritin, folate, vitamin B12, bilirubin, glucose, uremia, and C-reactive protein (CRP)—were available for a subset of children, depending on clinical assessment. Reticulocyte count and total iron binding capacity (TIBC) were not routinely measured. Other differential diagnoses, including zinc deficiency and bone marrow aspiration, were also not routinely assessed or performed. Sickle cell trait was identified through hemoglobin electrophoresis when available or when clinically justified by the pediatrician’s assessment.

Investigators carefully monitored data collection. To ensure data quality, double data entry verification was conducted, and the information from paper-based forms and electronic questionnaires was compared. Data cleaning was performed to verify frequencies, consistency, and missing values, and any errors identified were corrected.

### 2.5. Outcome Variable

The outcome variable for this study was the presence of anemia or the risk of developing anemia in children aged 6 months to 5 years. In the context of this study, children with hemoglobin (Hb) concentrations between 11.0 and 11.4 g/dL were classified as being in a borderline or pre-anemic stage—considered at elevated risk of developing anemia [6,11,22,23]. Hemoglobin cut-off values for childhood anemia classification were defined based on the recent WHO guidelines [52,53,54] and were stratified by age group as follows (Table 1):

Additional definitions relevant to this study are summarized in Table 2.

More details on definitions of anemia and the outcome variable can be found in the Supplementary File Table S1.

### 2.6. Exposure Variables

In this study, exposure variables included sociodemographic characteristics (sex, age, residence area, caregiver’s degree of kinship, caregiver’s education level, country of origin, and parental occupation) and nutritional characteristics (history of breastfeeding, complementary feeding, and intake of food groups). According to the WHO, minimum dietary diversity (MDD) is considered adequate when children aged 6–23 months consume at least five out of eight food groups in a 24 h period [61,62]. However, for children aged 24 months to 5 years, no official WHO threshold exists. Therefore, we applied an adapted cut-off of four or more food groups to define dietary diversity for children aged 6 months to 5 years, following criteria from previous studies [63,64,65,66]. In this study, food consumed the previous day was classified into the following seven food groups, based on WHO and Portuguese dietary guidelines [61,67]: (1) cereals and derivatives and tubers; (2) meat, fish, and eggs; (3) dairy products; (4) fruits; (5) legumes; (6) vegetables; (7) fats and oils. The dietary diversity score (DDS) was calculated as the number of food groups consumed during the previous day, with a DDS ≥ 4 considered “adequate” and <4 considered “inadequate” [63,64,65,66].

Exposure variables also included anthropometric characteristics (weight percentiles) [68] and other characteristics (vomiting or refusal to eat, presence of infections or inflammatory conditions, glucose and bilirubin levels, uremia, hospitalization, and duration of hospitalization). More details on exposure variables can be found in Supplementary File Table S1.

### 2.7. Data Analysis

Data were analyzed using SPSS 28.0 software (International Business Machine Corporation [IBM Corp] based in Armonk, New York, USA) [69]. Descriptive statistics, including frequency distributions and cross-tabulations, were performed to summarize the characteristics of study participants and key variables. As this was a purely descriptive study aimed at characterizing the profiles of children diagnosed with or at risk of anemia—without the inclusion of a control group—no inferential statistical analyses were conducted. As such, *p*-values were not reported, as the study did not assess statistical significance.

### 2.8. Ethical Considerations

This study was conducted in accordance with the Declaration of Helsinki and approved by the Ethics Committee for Health of the Central Lisbon University Hospital Center (CES-CHULC) (CES 947/2020, first approval on 19 November 2020; with the second approval extension on 3 April 2024). This research is also described as a sub-study that integrates the study protocol on the same topic (PAMC, Ref. Of 0110/CC/2020) approved by the Institutional Committee of Bioethics in Health of the Faculty of Medicine/Maputo Central Hospital (CIBS FM&HCM; HCM/004/2020, first approval on 30 March 2020, with the fourth approval extension granted on 29 February 2024).

## 3. Results

### 3.1. Sociodemographic Characteristics

**Figure 1:**
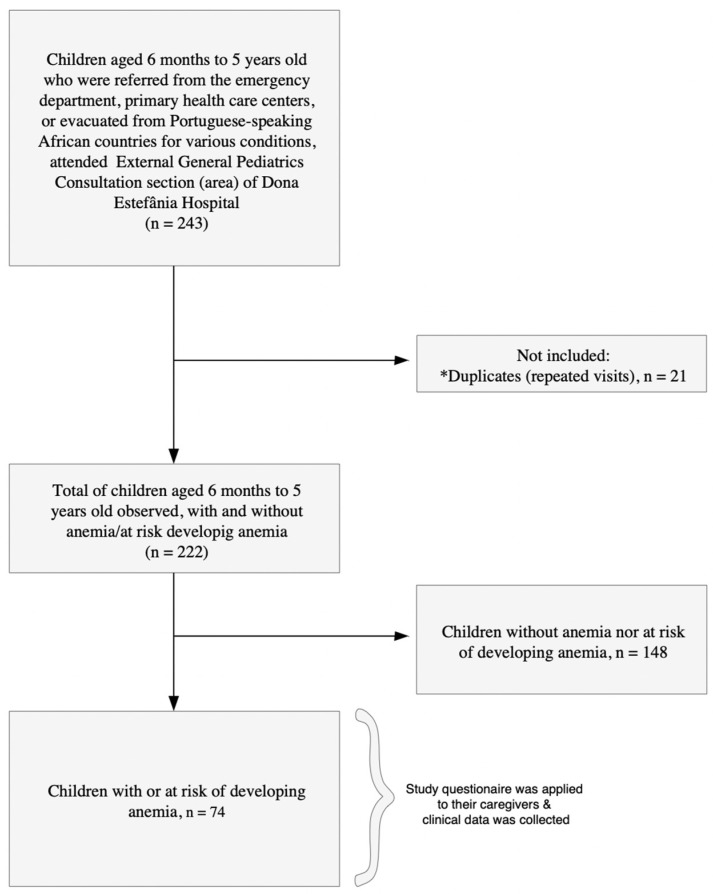
Flowchart of the study population. (*) Indicates repeated visits by children to the consultations.

A total of 222 children, aged 6 months to 5 years, who were referred from the emergency department, primary health care centers, or evacuated from Portuguese-speaking African countries for various conditions attended external general pediatric consultations between September 2023 and September 2024. Among them, 33.3% (74/222) children had anemia or were at risk of developing anemia, and their caregivers were invited to participate. All caregivers agreed to participate, resulting in a 100% acceptance rate (Figure 1). The reasons for consultation are detailed in Supplementary Files Tables S2 and S3. Among the 74 children, anemia-related reasons (including anemia combined with other diagnoses) accounted for 60.8% of consultations (45/74). Other clinical presentations (e.g., intestinal failure, abdominal pain, malformations) accounted for the remaining 39.2% (29/74).

Table 3 summarizes the sociodemographic characteristics of children with or at risk of developing anemia. Over half of the participants were male (42/74; 56.8%). The majority of children were aged 24 months to 5 years (46/74; 62.2%), lived in the Metropolitan Lisbon area (61/74; 82.4%), and were accompanied by their mothers to the consultations (63/74; 85.1%).

### 3.2. Childhood Anemia by Sociodemographic Characteristics

Out of the 74 children included in our study, 93.2% (69/74) were confirmed to be anemic or at risk of developing anemia based on hemoglobin (Hb) levels using age-specific cut-offs (Table 4). Five children (6.8%) had Hb values within the normal range. Although they did not meet the criteria for anemia based on Hb, these children exhibited altered red-cell indices, including microcytic (60.0%; 3/5) and normocytic (40.0%; 2/5) patterns (Supplementary File Table S4). Our results indicate that anemia cases in children aged 6 months to 5 years, measured by hemoglobin levels, were slightly higher in males (55.1%; 38/69) (Table 4). The majority of cases occurred in children residing in the Metropolitan Lisbon Area (82.6%; 57/69). Regarding caregiver characteristics, more than half of anemia cases were observed in children whose caregivers had a basic or secondary level of education (58.0%; 40/69). Children whose mothers were from CPLP countries (48.4%; 30/62) accounted for a higher proportion of cases of anemia compared to their peers.

### 3.3. Childhood Anemia by Nutritional and Clinical Characteristics

Table 5 presents the distribution of anemia cases—based on hemoglobin (Hb) levels—by nutritional and clinical characteristics among children aged 6 months to 5 years. The majority of anemic children had a history of exclusive breastfeeding (82.0%; 50/61) and had received or were currently receiving complementary feeding (85.5%; 59/69). A history of food selectivity behavior—such as refusal to eat certain foods or vomiting after consumption—was reported in 15.9% (11/69) of cases. Most anemic children (81.2%; 56/69) had an adequate dietary diversity score (DDS ≥ 4). Among children with anemia, 87.1% (27/31) had an adequate weight-for-age percentile. Additionally, elevated C-reactive protein (CRP) levels—suggestive of infection or inflammation—were present in 34.9% (15/43) of cases, while 65.1% (28/43) had normal CRP values.

### 3.4. Hematological and Iron Status Characteristics of Anemic Children

Table 6 presents the hematological and iron status characteristics of children who were anemic or at risk based on hemoglobin (Hb) levels. Two-thirds (63.1%; 41/65) of the children presented microcytic indices, particularly those aged 24 months to 5 years. Serum iron levels showed that 32.0% (8/25) of children were in the pre-anemic stage, and 40.0% (10/25) were anemic. Ferritin levels suggested iron deficiency anemia in 22.2% (4/18) of children, especially in those aged 24–59 months. Sickle cell trait was identified in a minority of cases (33.3%; 3/9), all within the older age group.

## 4. Discussion

This study characterized the profile of children aged 6 months to 5 years who attended the Dona Estefânia Hospital (HDE) in Lisbon, Portugal, and were diagnosed with or considered at risk of anemia. Our results showed that the overall prevalence of anemia among children in this age group was 33.3%, indicating that anemia poses a moderate public health concern [70,71]. Among those with anemia or at risk in our study sample, 93.2% exhibited either established anemia or altered red-cell indices, including microcytic and normocytic patterns, reflecting a substantial burden of disease within this subgroup. The prevalence reported in our study (33.3%) exceeds both the national (14.3%) and broader European region estimates (20.3–22.0%) [37,40]. It is also slightly higher than rates reported by other authors in various regions of Brazil (23.1%) [72] and in Timor-Leste (31.3%) [73]. However, it remains lower than those found in the Community of Portuguese Language Countries (CPLP), namely, in Brazil (38.1–56.6%) [74,75,76], Angola (44.4%) [77], Cape Verde (51.8%) [78], São Tomé and Príncipe (83.0%) [79], Equatorial Guinea (85.0%) [80], and in Mozambique (62.2–83.0%) [81,82,83].

The high rates of anemia reported in our study should be interpreted in the light of its unique sociodemographic characteristics. As such, these rates might be partly explained by the considerable portion of children with backgrounds from the CPLP and African regions, where anemia and hemoglobinopathies are more prevalent [84]. Additionally, these variations in anemia prevalence might also be attributed to a combination of geographical disparities (e.g., differences in climate), cultural factors (e.g., food practices), nutritional factors (e.g., dietary habits and food intake), genetic factors (e.g., inherited blood disorders), and socioeconomic context (e.g., caregivers’ education level and access to health facilities) [16,43,85,86,87].

### 4.1. Sociodemographic Patterns in Childhood Anemia

Our results indicate that anemia rates were slightly higher in male children (55.1%). Consistent with our findings, several studies [16,88,89,90] have shown that boys are generally more susceptible to anemia than girls. This may be attributed to increased prenatal and postnatal growth in males, which elevates their micronutrient requirements—often not fulfilled through diet alone [88,90].

Similar to previous studies [91,92,93], we found higher anemia rates (58.0%) among children whose caregivers had a lower level of education—a factor commonly associated with reduced health literacy and poorer health outcomes. In addition, the predominance of anemia in children whose mothers were from CPLP countries (48.4%) in our study sample reinforces the role of migration and socioeconomic disadvantage. Some studies [43] have shown that anemia plays a more significant role in disability and life imbalances in pregnant women and children under five years of age in CPLP countries than in Portugal. Nonetheless, in recent years, the burden of anemia and hemoglobinopathies in Portugal has been exacerbated by migration from CPLP countries [94]. Some authors [95] state that immigrant mothers often may carry pre-existing conditions common in their countries of origin (e.g., anemia and hemoglobinopathies) that are less frequent in host countries like Portugal. These women often request and receive less antenatal and postnatal care during and after pregnancy, affecting both their own health and that of their children.

In our study, the Metropolitan Lisbon Area (MLA) accounted for 82.6% of anemic cases. This aligns with a study [84] conducted in two of the MLAs (Amadora and Sintra), which reported high rates of anemia (30.1%) among migrant children and adolescents.

Thus, in line with previous studies [84], we emphasize the need for systematic hemoglobin screening among migrant children from African countries, where hemoglobinopathies are common, due to the importance of the disease in the Portuguese population. Broader socioeconomic strategies [96], nutrition education programs targeting caregivers with lower education levels [97], and strengthening the training of healthcare professionals in multicultural care [95] could be crucial for mitigating childhood anemia in this setting. Nationwide strategies for the prevention and management of anemia in Portugal, which integrate nutrition education, particularly for pregnant women and caregivers of young children [41,42], may play a vital role in addressing this public health issue. Further research on the relationship between socioeconomic disparities and childhood anemia is needed to inform future strategies and interventions.

### 4.2. Nutritional Characteristics in Childhood Anemia

In our study, we observed that 82.0% of anemic children had a history of exclusive breastfeeding up to six months of age, and 85.5% had received or were currently receiving complementary feeding. This highlights a critical window during the weaning period. This result can be explained by the fact that if exclusive breastfeeding occurs alongside low iron stores due to maternal anemia, it might not be sufficient to meet the growing iron requirements of the infant and can lead to iron depletion if not supplemented adequately after six months of age [10,11,98,99].

The quality and timing of complementary feeding may have influenced iron status, as the majority of anemia cases in our study sample (81.2%) occurred despite adequate dietary diversity scores (DDS ≥ 4). This finding suggests that dietary diversity alone may not adequately reflect iron intake, especially if iron-rich or fortified foods are not included in the child’s diet [83,98]. Food selectivity behaviors, reported in 15.9% of cases, may further contribute to inadequate iron intake. Although not predominant, these behaviors align with studies suggesting that feeding difficulties and sensory-related food aversions can hinder nutritional adequacy [15,16,100].

### 4.3. Clinical and Hematological Parameters in Childhood Anemia

Our findings showed that the majority of children exhibited microcytic red cell indices (63.1%), particularly among those aged 24 months to 5 years. This pattern is often caused by iron deficiency, which was corroborated by serum iron and ferritin levels in our study: 40.0% of children had iron-deficiency anemia based on serum iron, and 22.2% was based on ferritin. Approximately 33.3% of tested children carried the sickle cell trait. In line with previous studies [94], our results emphasize the importance of systematic hemoglobin screening among migrant children from African countries. In addition, 35.0% of anemic children had elevated C-reactive protein (CRP), suggesting that anemia of inflammation may contribute in about one-third of cases. This highlights the need for differential diagnosis, as inflammatory processes can mask iron-deficiency anemia and make difficult the interpretation of ferritin levels [12,13].

### 4.4. Strengths and Limitations

To our knowledge, this is the first hospital-based study in Lisbon to characterize children aged 6 months to 5 years who are diagnosed with or at risk of anemia. Given the limited existing research on this issue in Portugal, our study can serve as a baseline for future research (whether analytical cross-sectional or longitudinal) focused on the role of feeding practices, dietary habits, nutritional adequacy, and other potential influencing determinants, such as cultural, socioeconomic, and metabolic aspects, on childhood anemia in Portugal. In addition, by providing valuable evidence, our study establishes a foundation for improving the prevention, management, and care of anemia in children living in Portugal.

However, there are some limitations to our study. First, due to constraints from the COVID-19 pandemic, which placed significant pressure on the National Health Service (SNS) and pediatric care in Portugal, the data collection process was impacted. Consequently, this study adopted a purely descriptive design. Our one-year study focused on characterizing the profiles of children diagnosed with or at risk of anemia and did not include children without anemia, limiting the analysis of relationships or associations between various determinants and childhood anemia. Second, our sample universe was not randomly selected, as it consisted of all children aged 6 months to 5 years old who attended the external general pediatric consultations section of Dona Estefânia Hospital and were chosen based on availability during the study period. As such, our findings should not be generalized to all pediatric consultations or patient profiles at the hospital. Third, the sociodemographic composition of our sample included a substantial proportion of anemic children with backgrounds from the Community of Portuguese Language Countries (CPLP), reflecting the multicultural composition of the hospital’s catchment area. Our findings are especially relevant in providing recent evidence on childhood anemia within this specific context in Portugal. Consequently, they cannot be fully generalized to the general pediatric Portuguese population, and some comparisons presented with population-based studies should be interpreted with caution. Fourth, dietary needs differ substantially between a 6-month-old infant beginning complementary feeding and a 5-year-old child. While the WHO provides an age-appropriate threshold for children aged 6–23 months—defining adequate dietary diversity as the consumption of at least five out of eight food groups in the previous day—no official threshold exists for children aged 24 months to 5 years [61,62]. Therefore, similar to previous studies [63,64,65,66], we applied a single threshold across the entire 6-month to 5-year age range as a methodological simplification. This decision was also necessary due to the small sample size, which limited further stratification and has been acknowledged in the interpretation of our findings. Fifth, the lack of complete biochemical data (e.g., ferritin, serum iron levels, folate, vitamin B12), which are not routinely measured, and the cross-sectional descriptive nature of the study prevents causal inference. Nonetheless, the high participation rate and diverse sample might allow generalizability within similar urban pediatric populations in Portugal.

Therefore, future studies aiming at longitudinal monitoring, integrating qualitative data on caregiver beliefs and feeding practices, and exploring anemia associations with various determinants can contribute to providing more generalizable results and enhance our understanding of the factors influencing anemia in children in Portugal.

## 5. Conclusions

This study describes anemia among children aged 6 months to 5 years attending external general pediatric consultations in Portugal. Based on our findings, anemia poses a moderate public health concern among young children, particularly in populations where caregivers—especially mothers—have lower levels of education and are migrants. While dietary diversity was generally adequate, iron intake may still be insufficient, requiring further investigation.

Tailored public health strategies—including systematic screening, particularly among migrant children, caregiver education, and culturally sensitive care—are essential. Further research, including analytical cross-sectional or longitudinal studies, is necessary to better understand the role of feeding practices, dietary habits, nutritional adequacy, and other potential influencing determinants such as cultural, socioeconomic, and metabolic factors in childhood anemia in this setting.

## Figures and Tables

**Table 1:** Hemoglobin cut-off values for anemia in children aged 6 months to 5 years, stratified by age group.

Age Group	Anemia Status	Hemoglobin (Hb) Level (g/dL)
6–23 months	Not anemic	≥10.5
	Mild anemia	9.5–10.4
	Moderate anemia	7.0–9.4
	Severe anemia	<7.0
24 months to 5 years	Not anemic	>11.4
	Borderline/pre-anemic	11.0–11.4
	Mild anemia	10.0–10.9
	Moderate anemia	7.0–9.9
	Severe anemia	<7.0

**Table 2:** Anemia and iron status classification based on MCV, hematocrit, serum iron, and ferritin levels in children aged 6 months to 5 years.

Parameter	Age Group	Classification	Cut-Off/Range
MCV (fL)	6–23 months	Microcytic	<70
		Normocytic	70–86
		Macrocytic	>86
	24 months–5 years	Microcytic	<75
		Normocytic	75–87
		Macrocytic	>87
Hematocrit (%)	6–23 months	Not anemic	33.0–38.0
		Anemic	<33.0
	24 months–5 years	Not anemic	34.0–40.0
		Anemic	<34.0
Serum iron (mcg/dL)	All ages *	Not anemic	50.0–120.0
		Pre-anemic stage	30.0–50.0
		Anemic	<30.0
Ferritin (ng/mL)	All ages *	Not anemic	>30.0
		Iron deficiency anemia	12.0–30.0
		Risk of iron overload	>500.0
Notes	[28,29,52,53,54,55,56,57,58,59,60]		
	(*) Although serum iron levels may show minor physiological variations with age in children under five, in this study, standardized cut-offs were applied across the 6 months to 5 years group for consistency [59,60].		

**Table 3:** Sociodemographic characteristics of children aged 6 months–5 years at Dona Estefânia Hospital, PAMC, September 2023–September 2024.

Characteristic (n = 74)	Categories	N	%
Sex	Male	42	56.8
	Female	32	43.2
Child’s age	6 months–23 months	28	37.8
	24 months–5 years	46	62.2
Country of residence	Portugal	73	98.6
	Cape Verde	1	1.4
Region of residence	Metropolitan Lisbon Area (Greater Lisbon)	61	82.4
	Other regions (Setúbal peninsula, Alentejo, Madeira, West and Tagus Valley, etc.)	13	17.6
Caregiver’s Degree of Kinship	Mother	63	85.1
	Father	11	14.9
Caregiver’s Level of Education	Basic/Primary or Secondary Level	41	55.4
	Technical or Higher education (bachelor’s, master’s, doctorate)	22	29.7
	Other	11	14.9
Country of origin of the child’s mother	Portugal	20	29.9
	CPLP	32	47.8
	Other countries	15	22.4
Mother’s occupation (by role)	Specialized Intellectual and scientific roles	5	12.2
	Administrative, Managerial, or Support roles	36	87.8
Country of origin of the child’s father	Portugal	18	34.6
	Other countries	34	65.4
Father’s occupation (by role)	Administrative, Managerial, or Support roles	18	45.0
	Other roles	22	55.0
Notes:	CPLP: Community of Portuguese Language Countries. Mother’s other origin countries included Nepal, Bangladesh, Ukraine, Lithuania, Spain, India, Venezuela, Ivory Coast, and the Republic of Guinea (Conakry). Father’s other countries of origin included CPLP countries: Nepal, Bangladesh, India, and Ukraine.		
	Mother’s occupation, by roles, includes two large groups: Specialized Intellectual and Scientific roles (experts in intellectual and scientific professions) and Administrative, Managerial, or Support roles (administrative staff and similar, managers, self-employed individuals, entrepreneurs, service and sales staff, and unemployed or domestic workers).		
	Father’s occupation, by roles, includes two large groups: Administrative, Managerial, or Support roles (administrative staff and similar, managers, self-employed individuals, entrepreneurs, service and sales staff, and unemployed or domestic workers) and other roles (experts in intellectual and scientific professions, technicians and professionals at the intermediate level, and industrial, agricultural, and fishing workers).		

**Table 4:** Sociodemographic distribution of anemia cases and hemoglobin (Hb) levels among children aged 6 months to 5 years.

Characteristic	Category	Total with Anemia (N = 69)	6–23 Months ^1^ (n = 26)	24 Months–5 Years ^2^ (n = 43)
Sex	Male	38 (55.1%)	12 (46.2%)	26 (60.5%)
	Female	31 (44.9%)	14 (53.8%)	17 (39.5%)
Region of residence	Metropolitan Lisbon Area (Greater Lisbon)	57 (82.6%)	24 (92.3%)	33 (76.7%)
	Other regions (Setúbal peninsula, Alentejo, Madeira, West and Tagus Valley, etc.)	12 (17.4%)	2 (7.7%)	10 (23.3%)
Caregiver’s Degree of Kinship	Mother	58 (84.1%)	21 (80.8%)	37 (86.0%)
	Father	11 (15.9%)	5 (19.2%)	6 (14.0%)
Caregiver’s Level of Education	Basic/Primary or Secondary Level	40 (58.0%)	12 (46.2%)	28 (65.1%)
	Technical or Higher education (bachelor’s, master’s, doctorate)	19 (27.5%)	11 (42.3%)	8 (18.6%)
	Other	10 (14.5%)	3 (11.5%)	7 (16.3%)
Country of origin of the child’s mother	Portugal	18 (29.0%)	9 (36.0%)	9 (24.3%)
	CPLP	30 (48.4%)	9 (36.0%)	21 (56.8%)
	Other countries	14 (22.6%)	7 (28.0%)	7 (18.9%)
Mother’s occupation (by role)	Specialized Intellectual and scientific roles	3 (8.3%)	3 (18.8%)	0 (0.0%)
	Administrative, Managerial, or Support roles	33 (91.7%)	13 (81.3%)	20 (100.0%)
Country of origin of the child’s father	Portugal	15 (31.9%)	6 (30.0%)	9 (33.3%)
	Other countries	32 (68.1%)	14 (70.0%)	18 (66.7%)
Father’s occupation (by role)	Administrative, Managerial, or Support roles	16 (45.7%)	5 (33.3%)	11 (55.0%)
	Other roles	19 (54.3%)	10 (66.7%)	9 (45.0%)
Notes:	^1^ Hb < 10.5 g/dL—anemic ^2^ Hb ≤ 11.4 g/dL—anemic or at risk of anemia			
	CPLP: Community of Portuguese Language Countries. Mother’s other origin countries included Nepal, Bangladesh, Ukraine, Lithuania, Spain, India, Venezuela, Ivory Coast, and the Republic of Guinea (Conakry). Father’s other countries of origin included CPLP countries: Nepal, Bangladesh, India, and Ukraine.			
	Mother’s occupation, by roles, includes two large groups: Specialized Intellectual and Scientific roles (experts in intellectual and scientific professions) and Administrative, Managerial or Support roles (administrative staff and similar, managers, self-employed individuals, entrepreneurs, service and sales staff, and unemployed or domestic workers).			
	Father’s occupation, by roles, includes two large groups: Administrative, Managerial, or Support roles (administrative staff and similar, managers, self-employed individuals, entrepreneurs, service and sales staff, and unemployed or domestic workers) and other roles (experts in intellectual and scientific professions, technicians and professionals at the intermediate level, and industrial, agricultural, and fishing workers).			

**Table 5:** Distribution of anemia cases by nutritional and clinical characteristics among children aged 6 months to 5 years.

Characteristic	Category	Total with Anemia * (N = 69)	6–23 Months ^a^ (n = 29)	24 Months–5 Years ^b^ (n = 43)
Nutritional characteristics				
Exclusive breastfeeding history	Yes (Past/Present) ^c^	50 (82.0%)	20 (83.3%)	30 (81.1%)
	No	11 (18.0%)	4 (16.7%)	7 (18.9%)
History of complementary feeding	Yes (Past/Present) ^d^	59 (85.5%)	24 (92.3%)	35 (81.4%)
	No	10 (14.5%)	2 (7.7%)	8 (18.6%)
History of food selectivity behavior	Yes ^e^	11 (15.9%)	2 (7.7%)	9 (20.9%)
	No	58 (84.1%)	24 (92.3%)	34 (79.1%)
Dietary Diversity Score (DDS) ^f^	Adequate (DDS ≥ 4)	56 (81.2%)	23 (88.5%)	33 (76.7%)
	Inadequate (DDS <4)	13 (18.8%)	3 (11.5%)	10 (23.3%)
Supplements intake (post-anemia diagnosis) ^g^	Yes	35 (50.7%)	12 (46.2%)	23 (53.5%)
	No	34 (49.3%)	14 (53.8%)	20 (46.5%)
**Other characteristics**				
Weight percentile ^h^	Adequate for age	27 (87.1%)	14 (93.3%)	13 (81.3%)
	Inadequate for age	4 (12.9%)	1 (6.7%)	3 (18.8%)
C reactive protein (CRP)	Normal (CRP ≤ 5.0 mg/L)	28 (65.1%)	8 (47.1%)	20 (76.9%)
	High (CRP > 5.0 mg/L)	15 (34.9%)	9 (52.9%)	6 (23.1%)
Bilirubin	Normal (0.30–1.20 mg/dL)	10 (90.9%)	2 (66.7%)	8 (100.0%)
	Elevated (>1.20 mg/dL)	1 (9.1%)	1 (33.3%)	0 (0.0%)
Glucose	Normal (60–180 mg/dL)	18 (100.0%)	5 (100.0%)	13 (100.0%)
Urea	Uremia (Blood urea> 36.0 mg/dL)	5 (14.3%)	1 (7.7%)	4 (18.2%)
	Normal (5.0–36.0 mg/dL)	30 (85.7%)	12 (92.3%)	18 (81.8%)
Previous hospitalization	Yes	23 (34.3%)	9 (36.0%)	14 (33.3%)
	No	44 (65.7%)	16 (64.0%)	28 (66.7%)
Notes	^a^ Hb < 10.5 g/dL—anemic. ^b^ Hb ≤ 11.4 g/dL—anemic or at risk of anemia. ^c^ Refers to children who consumed only mother’s milk or formula milk up until 6 months of age (inclusively). ^d^ Refers to children who initiated complementary feeding at 6 months of age. ^e^ Refers to children whose mothers reported a history of food selectivity behavior (e.g., children who eat “normally” at kindergarten or school but refuse to eat the same foods at home), refusal to eat certain types of food (such as meat), or vomiting after consuming certain types of food (e.g., due to irritability or irritated behavior, abdominal pain, or an unspecified reason. ^f^ Dietary diversity score (DDS): number of food groups consumed during the previous day. A DDS ≥ 4 was considered “adequate”, and a DDS < 4 was considered “inadequate”. ^g^ Refers to iron or nutritional supplements intake after confirmation of anemia diagnosis during the period of the study. ^h^ Based on World Health Organization percentiles. Inadequate for age: percentile <3 (low weight for age) or percentile > 97 (high weight for age). ^(*)^ The number of cases may vary across categories due to missing responses for some variables. Percentages are calculated based on the number of valid responses.			

**Table 6:** Hematological and iron status characteristics of children anemic or at risk, by age group.

Characteristic	Total Anemic or at Risk of Anemia * (N = 65)	6–23 Months (n = 23)	24–59 Months (n = 42)
**Mean corpuscular volume (MCV) (fL) ^a^**			
Microcytic	41 (63.1%)	9 (39.1%)	32 (76.2%)
Normocytic	21 (32.3%)	13 (56.5%)	8 (19.0%)
Macrocytic	3 (4.6%)	1 (4.3%)	2 (4.8%)
**Hematocrit (%)** ^b^			
Normal	16 (24.6%)	5 (21.7%)	11 (26.2%)
Anemic	49 (75.4%)	18 (78.3%)	31 (73.8%)
**Serum iron (mcg/dL)** ^c^			
Not anemic	7 (28.0%)	2 (28.6%)	5 (27.8%)
Pre-anemic stage	8 (32.0%)	1 (14.3%)	7 (38.9%)
Anemic	10 (40.0%)	4 (57.1%)	6 (33.3%)
**Ferritin levels (ng/mL) ^d^**			
Not anemic	12 (66.7%)	6 (85.7%)	6 (54.5%)
Iron deficiency anemia	4 (22.2%)	0 (0.0%)	4 (36.4%)
Risk of iron overload	2 (11.1%)	1 (14.3%)	1 (9.1%)
**Sickle cell trait ^i^**			
No	6 (66.7%)	3 (100.0%)	3 (50.0%)
Yes	3 (33.3%)	0 (0.0%)	3 (50.0%)
Notes:	ᵃ For children aged 6–23 months, microcytic is defined as MCV < 70 fL, normocytic as 70–86 fL, and macrocytic as >86 fL. For children aged 24 months–5 years, microcytic is <75 fL, normocytic is 75–87 fL, and macrocytic is >87 fL. ᵇ Hematocrit levels below 33.0% (6–23 months) or 34.0% (24–59 months) are considered anemic. ᶜ Based on serum iron concentrations: not anemic = 50–120 mcg/dL; pre-anemic stage = 30–50 mcg/dL; anemic < 30 mcg/dL. ᵈ Based on ferritin levels: not anemic > 30 ng/mL; iron deficiency anemia = 12–30 ng/mL; risk of iron overload > 500 ng/mL. ^i^ Sickle cell trait was identified through hemoglobin electrophoresis. ^(*)^ Total N may vary due to missing data. Percentages are based on the number of valid responses per variable.		

## Data Availability

Data from this study are not publicly available due to patients’ privacy and ethical restrictions. However, the data presented in this study are available in this article and its Supplementary Information files.

## Supplementary Materials

The following supporting information can be downloaded at: https://www.mdpi.com/article/10.3390/children12070832/s1, Supplementary File Table S1. Study variables; Supplementary File Table S2. Reasons for consultation in anemic or at-risk children aged 6 months to 5 years at Dona Estefânia Hospital; Supplementary File Table S3. Reasons for consultation among children with a history of food selectivity, refusal to eat, or vomiting after eating; Supplementary File Table S4. Distribution of non-anemic children by age group, mean corpuscular volume, hematocrit, and iron status; Supplementary File Table S5. Distribution of anemia cases among children aged 6 months to 5 years by nutritional characteristics.

## Abbreviations

The following abbreviations are used in this manuscript:

AIDS	Acquired immunodeficiency syndrome
CBC	Complete blood count
CPLP	Community of Portuguese Language Countries
CRP	C-reactive protein
DDS	Dietary diversity score
ID	Iron deficiency
IDA	Iron deficiency anemia
Hb	Hemoglobin
HIV	Human immunodeficiency virus
HDE	Dona Estefânia Hospital
MCV	Mean corpuscular volume
MDD	Mininum dietary diversity
RCBs	Red blood cells
TIBC	Iron binding capacity
UI	Uncertainty interval
ULS	Unidade Local de Saúde
WHO	World Health Organization
