# Supplementary material for: Characteristics of Anemia in Children Aged 6 Months to 5 Years Attending External Consultations at a Pediatric Hospital in Lisbon, Portugal
Source: Children (Basel). 2025 Jun 24;12(7):832. doi: 10.3390/children12070832 (PMC12293216; doi:10.3390/children12070832)
Supplement: Supplementary file 1 [file children-12-00832-s001.zip › children-3649110-supplementary.pdf]

Supplementary file Table S1. Study variables.

Variables	Description
**Child Anemia**	
Anemia, ferropenia or mycrocitosis	Anemia status of alll children aged 6months-5 years. Categorized as No and Yes
Anemia or anemia risk, based on Hb level)	For children aged 6-23 months: Hb < 10.5 g/dL — anemic.<br>For children aged 24 months – 5 years: Hb ≤ 11.4 g/dL — anemic or at risk of anemia.
Anemia severity classification, based on Hb level	
Anemia, based on Hematocrit level	Hematocrit levels below 33.0% (6–23 months) or 34.0% (24–59 months) are considered anemic.
Anemia, based on size of red blood cell (RCB) measured by the mean corpuscular volume (MCV)	For children aged 6–23 months, microcytic is defined as MCV < 70 fL, normocytic as 70–86 fL, and macrocytic as > 86 fL. For children aged 24 months–5 years, microcytic is < 75 fL, normocytic is 75–87 fL, and macrocytic is > 87 fL.
Iron deficiency, based on serum iron level	Based on serum iron concentrations: not anemic = 50-120 mcg/dL; pre-anemic stage = 30-50 mcg/dL; anemic <30 mcg/dL.
Iron deficiency anemia (IDA), based on ferritin level	Based on ferritin levels: not anemic >30 ng/mL; iron deficiency anemia = 12-30ng/mL; risk of iron overload >500ng/mL.
Sickle-cell trait	Categorized as "No" and "Yes, sickle cell trait"
**Sociodemographic characteristics**	
Gender	Categorized as "Male" and "Female"
Child´s age	Categorized as "6 months-23 months", "24 months – 5 years"
Country of residence	Categorized as "Portugal" and "Other"
Region of residence	Categorized as "Great Lisbon" and "Other regions"
Caregiver's Degree of kinship	Categorized as "Mother" and "Father"
Caregiver´s Level of education	Categorized as "Basic or Secondary" and "Technical or Higher education"
Country of origin of the child´s mother	Categorized as "Portugal", "CPLP" and "Other countries"
Mother's occupation (by role)	Categorized as "Specialized Intellectual and scientific roles" and "Administrative, Managerial or Support roles"
Country of origin of the child's father	Categorized as "Portugal" and "Other countries"
Father´s occupation (by role)	Categorized as "Administrative, Managerial or Support roles" and "Other roles"

**Nutritional characteristics**	
Had exclusive breastfeeding	History of exclusive breastfeeding. Categorized as "No" and "Yes"
Complementary feeding	Categorized as "No" and "Yes"
Cereals and derivatives, tubers	Categorized as "No" and "Yes"
Meat, fish and eggs	Categorized as "No" and "Yes"
Dairy products	Categorized as "No" and "Yes"
Fruits	Categorized as "No" and "Yes"
Legumes	Categorized as "No" and "Yes"
Vegetables	Categorized as "No" and "Yes"
Fats and oils	Categorized as "No" and "Yes"
Dietary dietary diversity score	Dietary diversity score (DDS): number of food groups consumed during the previous day. A DDS ≥ 4 was considered as "adequate" and a DDS< 4 was considered "inadequate".
Supplements intake	Categorized as "No" and "Yes"
**Anthropometric characteristics**	
Weigh percentile	Categorized as "Adequate weight for age (Percentile 3-97)" and "Not adequate for age [Percentile <3 (low weight for age) or Percentile >97 (high weight for age]"
**Health characteristics**	
Food selectivity	Categorized as "No" and "Yes"
C reative protein (CRP) level	Categorized as " Normal (CRP ≤ 5.0 mg/L)" or "High (CRP > 5.0 mg/L) "
Glucose	Categorized as "Normal (60.0-180.0mg/dL)"
Bilirubin	Categorized as "Normal range (0.30-1.20mg/dL)" and "Elevated (>1.20 mg/dL)"
Urea	Categorized as "Normal (5.0-36.0mg/dL)" and "Uremia (>36mg/dL)"
Had any hospitalization (in the past)	Categorized as "No" and "Yes"

Supplementary file. Table S2. Reasons for consultation in anemic or at-risk children aged 6 months to 5 years at Dona Estefânia Hospital, PAMC, September 2023 – September 2024.

Reason for consultation*	6–23 months	24 months – 5 years	Total
Anemia	15 (53.6%)	19 (41.3%)	34 (45.9%)
Anemia (diagnosed in the context of previous hospital admission for vomiting)	0 (0.0%)	1 (2.2%)	1 (1.4%)
Anemia and Short stature for age	0 (0.0%)	1 (2.2%)	1 (1.4%)
Anemia and Feeding difficulty	1 (3.6%)	2 (4.3%)	3 (4.1%)
Anemia and Poor weight gain	1 (3.6%)	0 (0.0%)	1 (1.4%)
Anemia and Obesity	0 (0.0%)	1 (2.2%)	1 (1.4%)
Anemia and Food selectivity	1 (3.6%)	2 (4.3%)	3 (4.1%)
Anemia and Suspected Goldenhar Syndrome	0 (0.0%)	1 (2.2%)	1 (1.4%)
Abdominal pain	0 (0.0%)	1 (2.2%)	1 (1.4%)
Colon stenosis (sigmoid)	1 (3.6%)	0 (0.0%)	1 (1.4%)
Intestinal failure	1 (3.6%)	2 (4.3%)	3 (4.1%)
Fever with convulsions (resolved)	0 (0.0%)	1 (2.2%)	1 (1.4%)
Iron deficiency	1 (3.6%)	4 (8.7%)	5 (6.8%)
Gastroschisis	1 (3.6%)	0 (0.0%)	1 (1.4%)
Poor weight gain	1 (3.6%)	0 (0.0%)	1 (1.4%)
Congenital malformation (esophageal atresia)	1 (3.6%)	1 (2.2%)	2 (2.7%)
Congenital malformation (cloaca)	0 (0.0%)	1 (2.2%)	1 (1.4%)
Microcytosis	0 (0.0%)	1 (2.2%)	1 (1.4%)
Myelomeningoencephalocele	0 (0.0%)	1 (2.2%)	1 (1.4%)
Nephritis associated with IgA vasculitis	0 (0.0%)	1 (2.2%)	1 (1.4%)
Neutropenia associated with influenza A	1 (3.6%)	0 (0.0%)	1 (1.4%)
Obesity	1 (3.6%)	0 (0.0%)	1 (1.4%)
Constipation	1 (3.6%)	0 (0.0%)	1 (1.4%)
Intestinal obstruction	0 (0.0%)	1 (2.2%)	1 (1.4%)
Cerebral palsy	0 (0.0%)	1 (2.2%)	1 (1.4%)
Recurrent wheezing	0 (0.0%)	1 (2.2%)	1 (1.4%)
Down syndrome and heart disease	1 (3.6%)	0 (0.0%)	1 (1.4%)
Caudal regression syndrome	0 (0.0%)	1 (2.2%)	1 (1.4%)

Short bowel syndrome (SBS)	0 (0.0%)	2 (4.3%)	2 (2.7%)
Total	28 (100.0%)	46 (100.0%)	74 (100.0%)
Notes:	(*) This variable represents the main reason each children was referred from emergency services for evaluation or follow-up by a pediatrician.		

Supplementary file. Table S3. Reasons for consultation among children with a history of food selectivity, refusal to eat, or vomiting after eating, PAMC, September 2023 – September 2024

**Variables (N=12)**	**Categories**	**Refusal to eat/Food Selectivity**		**Refusal to eat/Vomiting after eating**		**Total (N=12)**	
		**N**	**%**	**n**	**%**	**n**	**%**
Reason for Consultation	Anemia	3	37,5	2	50	5	41,7
	Anemia and feeding difficulties	1	12,5	0	0	1	8,3
	Anemia and food selectivity	2	25,0	0	0	2	25
	Anemia and suspected Goldenhar Syndrome	1	12,5	0	0	1	8,3
	Abdominal pain	0	0,0	1	25	1	8,3
	Bowel failure	1	12,5	0	0	1	8,3
	Down syndrome	0	0,0	1	25	1	8,3

Supplementary file Table S4. Distribution of non-anemic children by age group, mean corpuscular volume, hematocrit, and iron status.

Characteristic	Total Non -Anemic (N= 5)	6-23 months (n=2)	24-59 months (n=3)
**Mean corpuscular volume (MCV) (fL)** [a]			
Microcytic	3 (60.0%)	0 (0.0%)	3 (100.0%)
Normocytic	2 (40.0%)	2 (100.0%)	0 (0.0%)
**Hematocrit (%)** [b]			
Not anemic	4 (100.0%)	1 (100.0%)	3 (100.0%)
**Serum iron (mcg/dL)** [c]			
Not anemic	2 (66.7%)	2 (100.0%)	0 (0.0%)
Pre-anemic stage	1 (33.3%)	0 (0.0%)	1 (100.0%)
**Ferritin levels (ng/mL)** [c]			
Not anemic	1 (100.0%)	1 (100.0%)	0 (0.0%)
Notes:	[a] For children aged 6–23 months, *microcytic* is defined as MCV < 70 fL, *normocytic* as 70–86 fL, and *macrocytic* as > 86 fL. For children aged 24 months–5 years, *microcytic* is < 75 fL, *normocytic* is 75–87 fL, and *macrocytic* is > 87 fL.<br>[b] Hematocrit levels below 33.0% (6–23 months) or 34.0% (24–59 months) are considered anemic.<br>[c] Although serum iron and ferritin values may exhibit minor physiological variations with age, standardized pediatric thresholds were applied uniformly for children aged 6 months to 5 years in this study [60,61<br>[d] The number of cases may vary across categories due to missing responses for some variables. Percentages were calculated based on the number of valid responses.		

Supplementary file Table S5. Distribution of anemia cases among children aged 6 months to 5 years by nutritional characteristics.

Characteristic	Category	Total with anemia (N= 69)	6-23 months [1] (n= 29)	24 months – 5 years [2] (n= 43)
History of exclusive breastfeeding[3]	Yes (Past/Present)	50 (82.0%)	20 (83.3%)	30 (81.1%)
	No	11 (18.0%)	4 (16.7%)	7 (18.9%)
History of complementary feeding[4]	Yes (Past/Present)	59 (85.5%)	24 (92.3%)	35 (81.4%)
	No	10 (14.5%)	2 (7.7%)	8 (18.6 %)
Dietary Diversity Score (DDS)[5]	Adequate (DDS ≥ 4)	56 (81.2%)	23 (88.5%)	33 (76.7%)
	Inadequate(DDS <4)	13 (18.8%)	3 (11.5%)	10 (23.3%)
Cereals and derivatives, tubers	Yes	55 (79.7%)	19 (73.1%)	36 (83.7%)
	No	14 (20.3%)	7 (26.9%)	7 (16.3%)
Meat, fish and eggs	Yes	60 (87.0%)	23 (88.5%)	37 (86.0%)
	No	9 (13.0%)	3 (11.5%)	6 (14.0%)
Dairy products	Yes	65 (94.2%)	23 (88.5%)	42 (97.7%)
	No	4 (5.8%)	3 (11.5%)	1 (2.3%)
Fruits	Yes	58 (84.1%)	25 (96.2%)	33 (76.7%)
	No	11 (15.9%)	1 (3.8%)	10 (23.3%)
Legumes	Yes	62 (89.9%)	26 (100.0%)	36 (83.7%)
	No	7 (10.1%)	0 (0.0%)	7 (16.3%)
Vegetables	Yes	51 (73.9%)	22 (84.6%)	29 (67.4%)
	No	18 (26.1%)	4 (15.4%)	14 (32.6%)
Fats and oils	Yes	14 (20.3%)	4 (15.4%)	10 (23.3%)
	No	55 (79.7%)	22 (84.6%)	33 (76.7%)
